# Involvement of miR775 in the Post-Transcriptional Regulation of Fructose-1,6-Bisphosphate Aldolase in Maize (*Zea mays* L.) Leaves Under Hypoxia

**DOI:** 10.3390/ijms26030865

**Published:** 2025-01-21

**Authors:** Dmitry N. Fedorin, Anna E. Khomutova, Alexander T. Eprintsev, Abir U. Igamberdiev

**Affiliations:** 1 Department of Biochemistry and Cell Physiology, Voronezh State University, 394018 Voronezh, Russia; rybolov@mail.ru (D.N.F.); anna.khomutova2002@gmail.com (A.E.K.); bc366@bio.vsu.ru (A.T.E.); 2 Department of Biology, Memorial University of Newfoundland, St. John’s, NL A1C 5S7, Canada

**Keywords:** fructose-1,6-bisphosphate aldolase, hypoxia, miR775, RNA interference, extracellular vesicles, *Zea mays* L.

## Abstract

Different types of microRNA participate in the post-transcriptional regulation of target genes. The content of several hypoxia-dependent miRNAs in plant cells, including miR775, increases in the conditions of oxygen deficiency. Electrophoretic studies of total RNA samples from the leaves of flooded seedlings of maize (*Zea mays* L.) revealed the presence of two interfering complexes with miR775 at 12 h of hypoxic incubation. A nucleotide sequence analysis of a sample containing the interfering complex of miR775 with mRNA from maize leaves showed a high degree of homology with the ICL/PEPM_KPHMT lyase family domain. It corresponded to a fragment of fructose-1,6-bisphosphate aldolase mRNA. By real-time PCR, we established the dynamics of the content of transcripts of aldolase isoenzyme genes under hypoxia in maize leaves. A decrease in the transcriptional activity of the aldolase 1 gene (*Aldo1*) correlated with a high content of miR775 in maize leaf cells. The fraction of extracellular vesicles sedimented at 100,000× *g*, was enriched with miR775. The accumulation of aldolase 2 (*Aldo2*) mRNA transcripts under hypoxic conditions indicates its participation in maintaining glycolysis when *Aldo1* expression is inhibited. We conclude that an increase in the total content of free miR775 and its participation in the suppression of the *Aldo1* gene represents an important mechanism in developing the adaptive reaction of cellular metabolism in response to hypoxia.

## 1. Introduction

Under hypoxic conditions, large-scale rearrangements of metabolic pathways take place in plants. In addition to processes such as the proteolytically mediated mechanism of O_2_ sensing or mitochondria-driven metabolic changes, other molecular mechanisms for the control of gene expression have recently been proposed as key regulators in the conditions of hypoxia and reoxygenation [1,2,3]. One such mechanism is the post-transcriptional regulation of target genes by corresponding miRNAs.

MiRNAs act as sequence-specific repressors of target gene expression either at the transcriptional level through DNA and histone methylation or at the post-transcriptional level through transcript cleavage or translational inhibition [4]. Unlike small interfering RNAs (siRNAs), miRNAs regulate the expression of multiple mRNAs due to different mechanisms of binding to the target sequence. MiRNAs are only partially complementary to their target mRNAs [5]. As a result, one miRNA strand can recognize a range of mRNAs and therefore have multiple targets [6].

In the cell, small RNAs function as part of protein complexes. MiRNAs can act through the RNA-induced silencing complex (RISC) or the RNA-induced transcriptional silencing complex (RITS). Several mechanisms have been proposed for the repression of translation initiation, including the dissociation of poly(A)-binding protein (PABP), promoting the disruption of the secondary 5′-cap structure and the inhibition of translation initiation [7], or the recruitment of translation inhibitors targeting eIF4E or eIF4G and the dissociation of eIF4A [8,9,10]. In the case of export to the cytoplasm, the RISC complex binds to one of the strands of the duplex, and the second strand is subsequently degraded. In the case of interaction with RITS, the formed complex acts in the nucleus, binding directly to DNA [11].

MiRNAs are independent mobile units, which are transported in the cell with the help of extracellular vesicles (EV) [12]. EVs are involved in the transmission of intercellular signals, and vesicle transport can potentially regulate plant responses to various environmental stress factors [13]. It was shown that EVs contained 10 miRNA families, i.e., these lipid structures can be considered as transport units of small non-coding RNAs in the process of forming an adaptive response under stress conditions [14].

Regulation at the molecular level directly affects the enzymatic system of metabolism. One of the most important metabolic pathways subject to changes under hypoxic stress conditions is carbohydrate metabolism [15]. Carbohydrate degradation via glycolysis in combination with enzymatic metabolism only partially compensates for the energy crisis associated with declining ATP levels but is still considered essential for survival in low oxygen conditions [16]. For example, in rice (*Oryza sativa* L.), starch availability plays a crucial role in survival after flooding. Rice seeds can germinate even in completely anoxic conditions, in particular, due to their ability to efficiently degrade starch in the absence of oxygen, while other cereals are unable to induce α-amylases under anoxic conditions [17,18].

The post-transcriptional regulation of glycolysis can be provided by miRNA. Numerous studies have shown the role of various miRNAs in the regulation of glycolytic processes in the microenvironment of cancer tumors [19,20]. MiRNAs regulating glycolysis under stress conditions have been identified also in plants. In particular, miR164c, miR169h, and miR395a can mediate the tricarboxylic acid (TCA) cycle, glycolysis, and pentose phosphate pathway in response to low potassium stress [21]. In conditions of oxygen deficiency, aerobic respiration, and photosynthesis are suppressed, which depletes the cellular ATP level within minutes. Glycolysis becomes the main way to synthesize ATP, but to maintain it, the regeneration of NAD(P)^+^ is necessary. The reoxidation of NAD(P)H occurs during lactic and ethanolic fermentations, which are stimulated by low oxygen levels [22]. Also, transaldolase and transketolase reactions allow the cell to control the flow of carbon between the oxidative pentose phosphate pathway and glycolysis [23]. Glucose-6-phosphate dehydrogenase plays an important function in modulating redox homeostasis when plants are exposed to abiotic stresses [24].

One of the first characterized non-conserved microRNAs in *A. thaliana* was miR775 [25]. It was shown to be induced by hypoxic conditions caused by flooding [26,27] and is involved in the regulation of cellular carbohydrate metabolism [28]. However, the functional role of miR775 in hypoxia and its target genes in maize requires further study. Recently, we established that miRNAs participate in phytochrome signal transduction under changing light conditions [29]. In this work, we studied the miR775A having in its composition the combination of five polymorphic nucleotides CGUAC. We are demonstrating that the increase of content miR775A in the conditions of oxygen deficiency caused by the full submergence of maize plants results in the formation of the interfering complex with mRNA having a high degree of homology with the domain corresponding to the fructose-1,6-bisphosphate aldolase mRNA. We conclude that the inhibition of fructose-1,6-bisphosphate aldolase expression in maize leaf cells via RNA interference with miR775A regulates the glycolytic pathway at the post-transcriptional level.

## 2. Results

### 2.1. Identification of miR775 Targets

**Figure 1:**
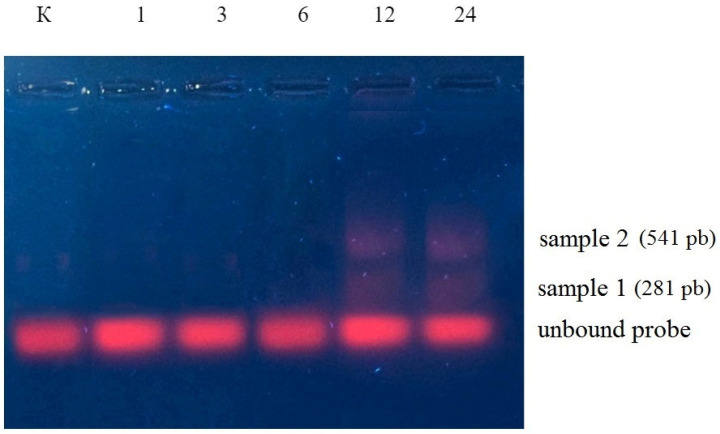
Electrophoretic study of the formation of fluorescent mRNA complex from maize leaves with the miR775A-ROX probe at different hours of hypoxic stress.

**Figure 2:**
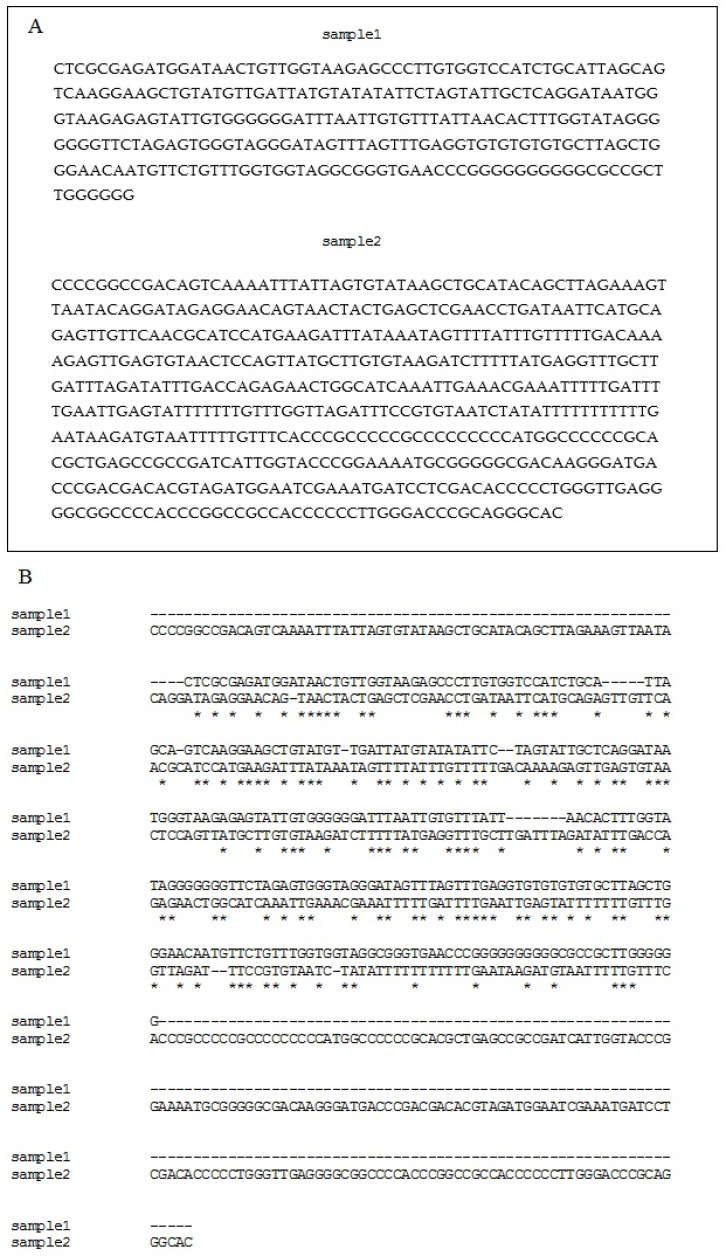
Nucleotide sequences of Sample 1 and Sample 2 (**A**) and their comparison (**B**). *—nucleotide match.

To identify miR775 targets, total RNA was reverse transcribed, and the resulting cDNA was incubated with a specific fluorescent probe miR775A−ROX. The electrophoretic study revealed that, up to 6 h exposure, one target was detected for miR775A, while, after 12 h, two targets were identified (Figure 1). The results of the hybridization analysis indicate a change in the number and type of target for miR775A in maize leaves during the development of hypoxic stress in the conditions of full submergence of the plants, since fluorescent complexes with the miR775A-ROX probe differ in size, which was established using DNA markers. To identify mRNAs that form interfering complexes with miR775A, these complexes were extracted and sequenced. The sequencing results showed that the total sample for mRNA from 1 to 24 h exposure had a similar nucleotide sequence, the size of which was 281 nucleotides (Figure 2).

After 12 h hypoxia, the formation of a second interfering complex was observed, the sequencing of which indicated a difference in nucleotide composition from the total for mRNA at 6 and 12 h. The size of the resulting sequence was 541 nucleotides.

The comparison of the obtained sequences of samples 1 and 2 of interfering complexes with miR775A from maize leaves at 6 and 12 h using the built-in BLAST module with nucleotide sequences of the MaizeGDB database (Maize Genetics and Genomics Database) allowed us to establish the correspondence of the obtained mRNA sequences.

**Figure 3:**
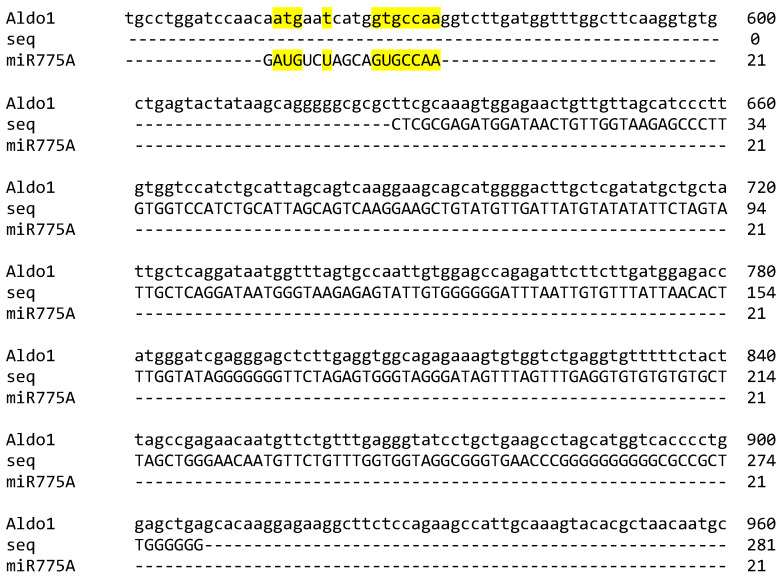
Determination of the site of attachment of miR775A to the nucleotide sequence of maize aldolase 1 mRNA (LOC100286050).

The highest match to the obtained sequence of Sample 1 was shown for the mRNA sequence of fructose-1,6-bisphosphate aldolase (LOC100286050) (Figure 3). The site of miR775A binding corresponds to the lyase domain region, confirming the possibility of forming an interfering complex with it (Figure 3). The analysis of the results of the correspondence between complex 1 and the nucleotide sequence of aldolase mRNA indicates their incomplete correspondence, which is a characteristic feature of miRNA. The binding of miRNA to cognate mRNAs usually leads to either the destabilization or suppression of the translation of target mRNAs. This is mainly due to the fact that the full length of a miRNA is almost never completely complementary to the MRE (miRNA recognition element). As a first rule, the pairing of approximately 6–8 nucleotides of the 5′ end of the miRNA, known as the “core” sequence, is generally considered necessary and sufficient for the functional formation of RISC [30,31]. MiR775A is characterized by a complementary interaction between the 3′ and 5′ ends, in particular, the formation of a paired element of 7 nucleotides at the 5′ end (Figure 3), which confirms its participation in the formation of the interfering complex.

**Figure 4:**
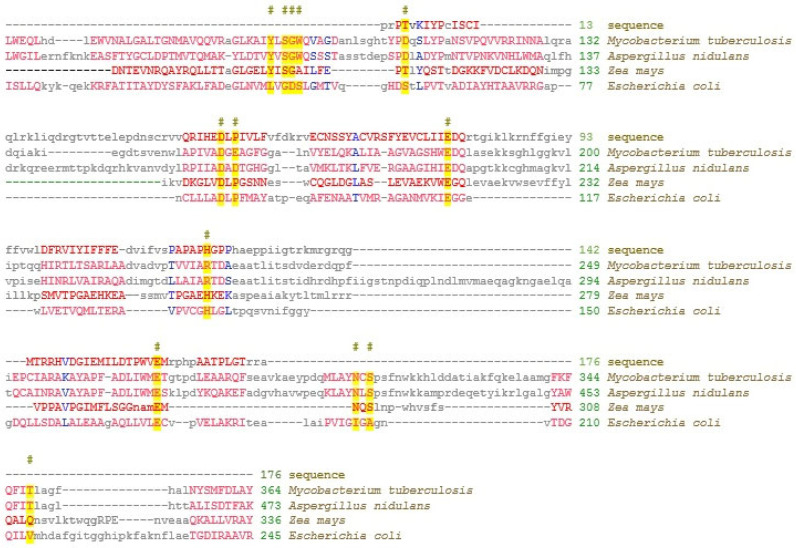
Comparative analysis of amino acid sequences of annotated proteins possessing the ICL/PEPM_KPHMT lyase domain and a fragment of the amino acid sequence based on the sequence of the RNA-interfering complex with maize miR775A. The amino acids (highlighted in gray) that form this domain are indicated on the sequences. #—correspondence of functional amino acids of the lyase domain.

The sequence of Sample 1 corresponds to a fragment of the domain sequence characteristic of members of the ICL/PEPM_KPHMT lyase superfamily of enzymes that form and cleave PC or CC bonds (Figure 4).

The obtained sequence of Sample 1 was translated using the program for translating the nucleotide sequence in the selected reading frames (http://molbiol.ru/scripts/01_13.html, accessed on 18 January 2025). Based on the obtained amino acid sequence, the amino acid composition with the domain structure of ICL/PEPM_KPHMT lyases was analyzed. Using the RPS-BLAST software v3.21 from NCBI (https://www.ncbi.nlm.nih.gov/Structure/cdd/docs/cdd_news.html, accessed on 18 January 2025), search studies of the database were carried out for compliance with the domain elements of annotated proteins based on the architecture of the ICL/PEPM_KPHMT lyase domain [32]. In the analyzed sequence, characteristic elements of this domain were identified, forming the corresponding active site of the enzyme, as well as the corresponding environment of amino acid residues, ensuring its correct orientation in the protein globule. Typical members are phosphoenolpyruvate mutase (PEPM), phosphonopyruvate hydrolase (PPH), fructose bisphosphate aldolase (ALDOA), carboxyphosphoenolpyruvate mutase (CPEP mutase), oxaloacetate hydrolase (OAH), isocitrate lyase (ICL), 2-methylisocitrate lyase (MICL), and ketopantoate hydroxymethyltransferase (KPHMT).

The nucleotide sequence analysis of Sample 2 of the miR775A interfering complex with maize leaf mRNA showed a high degree of homology with the *N*-acyltransferase domain, which is part of various enzymes that catalyze the transfer of an acyl group to a substrate. The highest affinity of the obtained sequence was shown with the mRNA of glycerol-3-phosphate acyltransferase (LOC100285746) [33]. The members include *N*-acetyltransferases (GNATs) such as aminoglycoside *N*-acetyltransferases, histone *N*-acetyltransferase (HAT) enzymes, and serotonin *N*-acetyltransferases that catalyze the transfer of an acetyl group to a substrate, arginine/ornithine *N*-succinyltransferase, myristoyl-CoA:protein *N*-myristoyltransferase, etc. [32].

**Figure 5:**
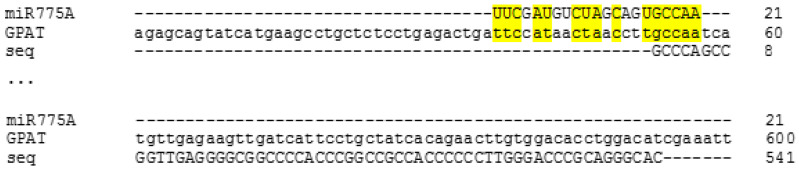
Nucleotide sequences of Sample 2 of miR775A and maize glycerol-3-phosphate acyltransferase (GPAT) mRNA with the miR775A attachment site indicated.

It was shown that the site of miR775A attachment corresponds to the lyase domain zone, which confirms the possibility of forming an interfering complex with it (Figure 5).

### 2.2. Aldolase Genes Aldo1 and Aldo2 Transcript Is the Target of miR775A

Using RPS-BLAST software from NCBI, it was found that miR775A forms an interfering complex with the mRNA of cytoplasmic aldolase isoenzyme in maize leaves. The degree of the homology of the sequence and mRNA of aldolase isoenzymes 1 (Aldo1) and 2 (Aldo2) was 78.3% and 46.1%, respectively. It was also found that the *Aldo1* gene is localized in chromosome 8, and *Aldo2* in chromosome 3.

**Figure 6:**
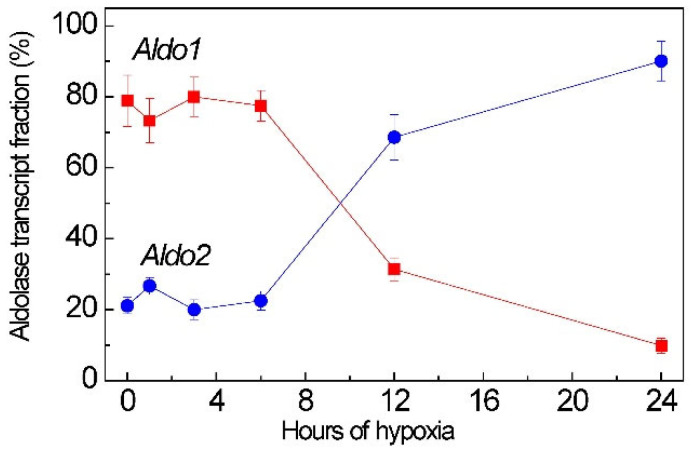
Relative levels of aldolase isoenzyme genes *Aldo1* (red squares) and *Aldo2* (blue circles) transcripts in maize leaves under hypoxia. The expression of the elongation factor ef-1ά reference gene revealed no statistically significant changes during the experiment.

Under normoxia, *Aldo2* is slightly involved in glycolysis (21% of the total transcript level of all cytoplasmic aldolase genes). However, under hypoxia, the indicators change significantly. At 24 h, the level of *Aldo2* transcripts is 90% of the total level of cytoplasmic aldolase transcripts (Figure 6). This suggests that under long-term hypoxia, *Aldo2* is the priority isoenzyme of cytoplasmic aldolase in maize leaves.

### 2.3. Aldolase and Glucose-6-Phosphate Dehydrogenase Activities Under Hypoxic Conditions

**Figure 7:**
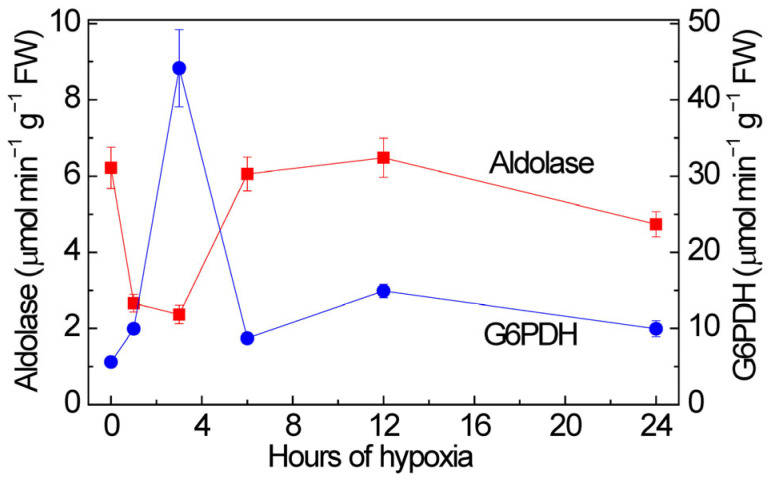
Total activity of fructose-1,6-bisphosphate aldolase (red squares) and glucose-6-bisphosphate dehydrogenase (G6PDH) (blue circles) in maize leaves under hypoxia.

The regulation of the expression of aldolase isoenzymes by miR775A is reflected in the overall enzyme activity during hypoxia (Figure 7). In the first 3 h, a gradual decrease in enzyme activity is observed, but after 6 h, a gradual return of activity to a value close to the initial level occurs. Based on the obtained results, it can be assumed that the *Aldo1* is blocked at the post-transcriptional level by miR775A, but to maintain glycolysis, *Aldo2* mRNA accumulates, and the second isoenzyme of cytoplasmic aldolase partially compensates for the blocking of the main isoenzyme to maintain glycolysis.

The measurement of the total activity of glucose-6-phosphate dehydrogenase showed that, under anaerobic conditions, the activity of this enzyme gradually increases, reaching its maximum at 3 h (Figure 7). At 6 h, there is a sharp decrease in the activity of glucose-6-phosphate dehydrogenase and the activity level remains close to the initial value. A sharp decrease in the activity of this enzyme at 6 h can be explained by competition with Aldo2, which subsequently compensates for blocking the main isoenzyme of the cytoplasmic aldolase.

### 2.4. Participation of Extracellular Vesicles in miR775A Transport

**Figure 8:**
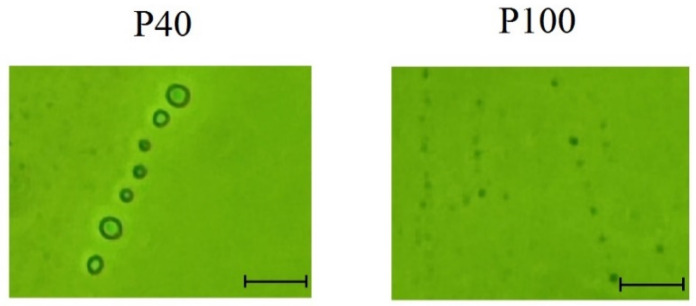
Photographs of extracellular vesicle fractions P40 and P100 taken using an Olympus CX41 microscope at 1000× magnification. P40 is the vesicle fraction obtained after differential centrifugation at 40,000× *g* and having a size of >170 nm. P100 is the vesicle fraction isolated after centrifugation at 100,000× *g* and having a size of up to 147 nm. The bar corresponds to 1000 nm (1 µm).

Differential ultracentrifugation was used to obtain extracellular vesicles of fractions P40 (40,000× *g*) and P100 (100,000× *g*) from maize leaves. Microscopy showed that fraction P40 was dominated by larger vesicles measuring 170 to 658 nm, while fraction P100 contained vesicles of the size 87 to 147 nm (Figure 8). Thus, differential ultracentrifugation allowed us to isolate two fractions of extracellular vesicles, the distinguishing feature of which is their size. By ultracentrifugation at 40,000× *g*, larger particles measuring over 170 nm were isolated.

The content of miR775A was analyzed in extracellular vesicles isolated from maize leaves using real-time PCR. It was found that different fractions of vesicles showed differences in the content of the studied miRNA. MiR775A was practically absent in the vesicles of the fraction P40 (0.57% of the total amount in the studied tissue). At the same time, the presence of miR775A was detected in the vesicles of fraction P100 (99.43%). Based on the data of quantitative PCR with cDNA obtained from green maize leaves, it can be concluded that the main amount of microR775A was detected in extracellular vesicles of the fraction P100.

**Figure 9:**
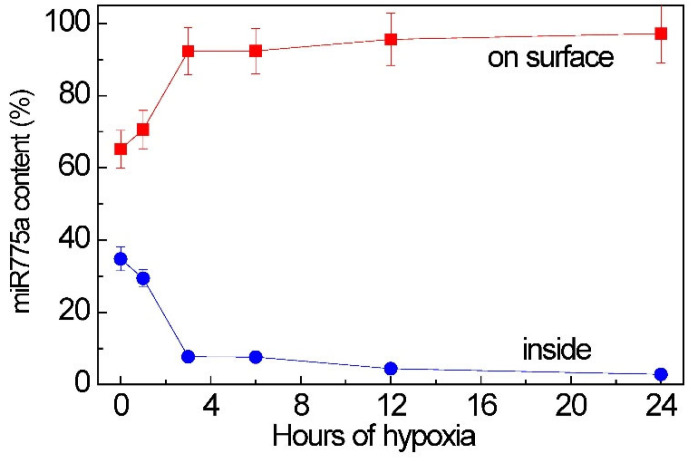
The relative content of miR775A on surface and inside the P100 vesicles in maize leaves under hypoxia.

By using the inhibitor analysis, we identified a difference in the quantitative distribution of miR775A within the P100 fraction under normal conditions and during hypoxia, with their predominant location on the vesicle surface. The proportion of miR775A located inside extracellular vesicles was no more than 34% of its total amount associated with the P100 fraction vesicles. Real-time PCR showed a change in the amount of miR775A located on the surface of P100 vesicles and inside them, as hypoxic stress developed, increasing this indicator (Figure 9). Consequently, most of the miR775A is located on the surface of extracellular vesicles, associating with the AGO1 protein, which protects them from proteases [34].

## 3. Discussion

It was previously established that miR775A is a hypoxia-dependent miRNA, as evidenced by a 3.25-fold change in its amount under anaerobic stress. Previous studies have also shown that this miRNA acts through RNA interference by binding to the RISC complex and regulates target genes at the transcriptional level [35].

Aldolase (FBA, EC 4.1.2.13) is one of six non-regulated glycolytic enzymes (its activity is regulated not by effectors or posttranslational modification, but by the regulation of protein expression or degradation) and catalyzes the reversible conversion of fructose-1,6-bisphosphate to glyceraldehyde-3-phosphate and dihydroxyacetone phosphate. The cytosolic forms of the enzyme are involved in the glycolytic pathway and gluconeogenesis [36], and chloroplast forms have also been identified.

In recent years, considerable attention has been paid to the role of FBAs in the regulation of plant growth and development. FBA isozymes have been identified in photosynthetic tissues, one of which is localized in the cytosol (cFBA) and the other in chloroplast (pFBA). Among the eight isozymes in Arabidopsis, three of them (AtFBA1–3) have a high similarity with FBAs in the chloroplast, and five (AtFBA4–8) correspond to FBAs localized in the cytoplasm. In tomatoes, SlFBA1–5 and SlFBA6–8 proteins were predicted to be localized in the chloroplast and cytoplasm, respectively [37]. In maize, two major anaerobic proteins have been identified, ANP35.5 and ANP33A, corresponding to cytoplasmic fructose-1,6-bisphosphate aldolase [38].

There is reason to assume that RNA interference with miR775A during long-term hypoxia (Figure 3) blocks the expression of the *Aldo1* gene at the post-transcriptional level. As a result, glycolysis is gradually inhibited. However, the glycolytic pathway operation remains important under hypoxic conditions, although not as intensively as under normoxia. The glycolytic pathway functions under hypoxic conditions, although not as intensively as under normoxia, since the rate of pyruvate synthesis during hypoxia must be reduced in order not to activate respiratory oxygen consumption [39]. As a result, glycolysis is gradually inhibited during the first hours of hypoxia. The content of glucose-6-phosphate and fructose-6-phosphate increased in the leaves of Arabidopsis and soybean under hypoxic conditions for the first 3 h of hypoxia, but the prolongation of oxygen deficiency (6 h) caused a drop in their concentration below the control [40]. Aldo2 can catalyze the aldolase reaction during the development of hypoxia, as evidenced by the accumulation of mRNA transcripts of this isoenzyme during the exposure of plants to oxygen deficiency (Figure 4). Changes in the transcriptional activity of the *Aldo1* and *Aldo2* genes in maize under hypoxia may provide a coordinated replacement of aldolase isoenzymes from Aldo1 to Aldo2 to ensure the maintenance of glycolysis under oxygen deficiency.

In plant cells, under hypoxia, ATP production in the mitochondria hampered the oxidation of NADH to NAD^+^, which is suppressed in the mitochondrial electron transport chain. This leads to the accumulation of NADH and NAD^+^ deficiency, which can ultimately lead to the inhibition of glycolysis and the tricarboxylic (TCA) acid cycle, where it is required as a cofactor [41,42]. The use of pyrophosphate (PPi) as an alternative to ATP is another way to maintain cellular energy homeostasis, but the use of the PPi pool does not provide long-term adaptation [43,44]. An alternative to maintaining carbohydrate metabolism under hypoxia is the activation of the oxidative pentose phosphate pathway, which is in good agreement with our data on the mechanism of aldolase activity suppression by RNA interference in the first 6 h of hypoxic conditions. The inhibition of this enzyme leads to the accumulation of fructose-6-phosphate [24,45].

We have shown that miR775A regulates glycolysis by adjusting the operation of different aldolase isoenzymes, but microRNAs can also act as an intercellular signaling molecule and regulate metabolism in other plant tissues. Previously, the content of miR775A was analyzed in extracellular vesicles isolated from maize leaves using real-time PCR. It was found that miR775A is contained only in vesicles of the P100 fraction [35].

Plants under stress use multiple gene regulation mechanisms to restore cellular homeostasis, including regulation at the post-transcriptional level [42]. Under hypoxia, the most sensitive process to changes in oxygen levels in plant cells is mitochondrial respiration. The disruption of its operation leads to changes in the general metabolism and cellular energy status. Many miRNAs are involved in the adaptive reorganization of metabolism to anaerobic conditions [26,46]. Changes in the miR775A level under oxygen deprivation allow us to classify it as a hypoxia-dependent miRNA [26]. The assessment of RNAi complex formation using a specific ROX-containing probe showed that miR775A acts through the RNAi pathway by binding to the RISC complex and post-transcriptionally interacts with its complementary mRNA [35].

To identify the type of mRNA that forms interfering complexes with miR775A, these complexes were extracted and sequenced. Their analysis indicates that miR775A does not fully correspond to the nucleotide sequence of fructose-1,6-bisphosphate aldolase mRNA. Using RPS-BLAST software from NCBI, it was possible to find out that the site of miR775A binding corresponds to the lyase domain zone, which confirms the possibility of forming an interfering complex with it.

It was also possible to determine that miR775A forms an interfering complex with the mRNA of cytoplasmic aldolase isoenzymes in maize leaves. The highest percentage of sequence and mRNA homology belongs to the Aldo1 isoenzyme. It is the main isoenzyme of cytoplasmic fructose-1,6-bisphosphate aldolase in the Embden-Meyerhof-Parnas glycolytic pathway. It was also possible to record the accumulation of *Aldo2* mRNA transcripts, which indicates its participation in maintaining glycolysis under hypoxic conditions when the main isoenzyme of cytoplasmic aldolase is blocked. This is confirmed by the obtained data on changes in the total aldolase activity during hypoxia. Also, an increase in the activity of the key enzyme of the oxidative pentose phosphate pathway—glucose-6-phosphate dehydrogenase—in the first 6 h of hypoxia indicates the activation of this metabolic pathway during short-term hypoxia.

A number of hypoxia-dependent miRNAs (miR159, miR166) were found in the composition of extracellular vesicles [47], which indicates the role of these miRNAs as signaling mobile units under low oxygen conditions. The presence of hypoxia-dependent miR775a in the P100 vesicles confirms its participation in plant adaptation to flooding as an intercellular signaling molecule, also acting as a regulatory element in the composition transported by extracellular vesicles. Changes in the amount of miR775A located on the surface of P100 vesicles with the development of hypoxic stress towards an increase in this indicator indicate its active synthesis. The accumulation of miR775A and an increase in its amount on the surface of vesicles during hypoxia leads to a gradual degradation of the biosynthesis of Aldo1, and the induction of the second isoenzyme of cytoplasmic fructose-1,6-bisphosphate aldolase (Aldo2) and the restoration of the indicators of the total activity of fructose-1,6-bisphosphate aldolase after 6 h of hypoxia.

**Figure 10:**
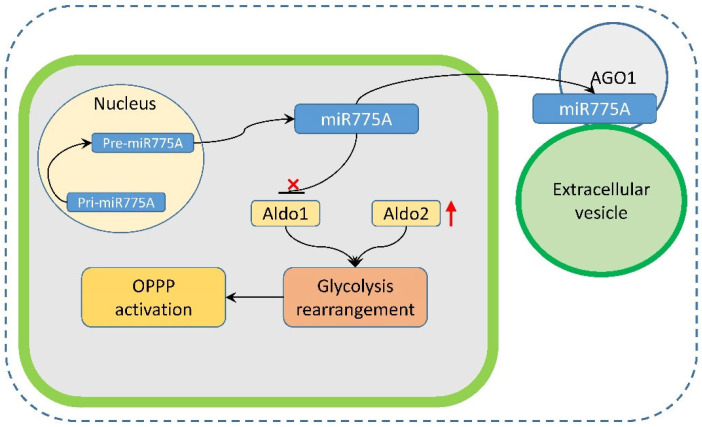
Hypothetical scheme of the participation of miR775A in the stress response of plants under hypoxic conditions. The microRNA is initially transcribed from the precursor gene as a long primary transcript (pri-miR775A), which is processed into the hairpin-shaped precursor (pre-miR775A), and then processed into miR775A. It is transferred from the cell and in association with the AGO1 protein becomes attached to the extracellular vesicle participating in the intercellular communication. It inhibits the expression of the Aldo1 isozyme, which leads to the activation of the expression of the Aldo2 isoenzyme, the rearrangement of glycolysis, and the activation of the oxidative pentose phosphate pathway (OPPP).

The results of this study on the analysis of the content and location of miR775A in extracellular vesicles showed that they can transfer miR775A, performing the function of intercellular communication. The transport of the analyzed miRNA can represent an important mechanism of intercellular signaling (Figure 10) in the development of an adaptive reaction of cellular metabolism in response to hypoxia.

## 4. Material and Methods

### 4.1. Object of Investigation

Leaves of 14-day-old maize (*Zea mays* L., cv. Voronezhskaya-76), grown hydroponically with 12 h daylight of intensity 90 μmol quanta m^−2^ s^−1^ at 25 °C, were used in this study.

### 4.2. Creating Hypoxic Conditions

The effect of low oxygen concentrations in the environment was performed by submerging maize plants with the root system in distilled water [48,49,50]; the plants were kept completely submerged for 24 h. The plants placed in a desiccator without immersion in water were used as a control group. To exclude the influence of photosynthesis, both groups of plants were preliminarily exposed to the dark for 24 h before the experiment. Throughout the entire experiment, the plants were in conditions without light sources.

### 4.3. Isolation of Total mRNA

The isolation of total RNA from plant samples was performed by guanidine thiocyanate-phenol-chloroform extraction [51]. LiCl was used as a precipitant [52].

### 4.4. Analytical Electrophoresis of Nucleic Acids

A qualitative analysis of RNA was performed by electrophoretic separation in 1% agarose gel. Ethidium bromide was used as a dye.

### 4.5. Reverse Transcription

Reverse mRNA transcription was performed using M-MuLV reverse transcriptase (SibEnzyme, Novosibirsk, Russia) according to the manufacturer’s instructions. To obtain the cDNA of total cellular RNA, Oligo(dT)15 primer (Eurogen, Moscow, Russia) was used. The parameters for performing reverse transcription were as follows: incubation of the mixture at 70 °C for 5 min, 37 °C for 60 min, and 70 °C for 10 min.

To obtain the cDNA of the analyzed miRNA, reverse transcription was performed with a specifically developed probe for miR775A [53]. The parameters for performing reverse transcription were as follows: the incubation of the mixture at 16 °C for 30 min, 42 °C for 30 min, and 85 °C for 5 min.

### 4.6. Real-Time Polymerase Chain Reaction

The polymerase chain reaction with gene-specific primers was performed using the AmpliSence reagent kit (Helikon, Moscow, Russia). The real-time PCR was performed on a LightCycler 96 instrument (Roche, Solna, Sweden). The reference gene was the elongation factor ef-1ά gene [54]. The nucleotide composition of the primers was as follows:

– for *Aldo1* (LOC100286050): forward—5′-AAGCCCGAAGACACCGATCT-3′; reverse—5′-AAGCAACAGATTTCGCGGTG-3′;
– for *Aldo2* (LOC100272913): forward—5′-GTGCCAACAACCTCTACGT-3′; reverse—5′-TCTGTTGTGTTGGCACAGG-3′.

The amplification parameters were as follows: preliminary denaturation—95 °C for 5 min, cycle—95 °C for 30 s, 58 °C for 30 s, and 72 °C for 30 s (detection), and final elongation—72 °C for 10 min.

The quantitative assessment of mature miR775A by qPCR was performed using a specific stem-loop RT-qPCR probe [53] with the formation of the 3′ priming site of the HSV-1 sequence. The nucleotide composition of miR775A primers was as follows: forward—5′-CACTGATTCGATGTCTAG-3′; reverse—5′-GTGCAGGGTCCGAGGT-3′. The amplification parameters were as follows: pre-denaturation—95 °C for 5 min, cycle—95 °C for 30 s, 58 °C for 30 s, and 72 °C for 30 s (detection), and final elongation—72 °C for 10 min.

Opticon MonitorTM Software v.3.1 (Bio-Rad, Hercules, CA, USA) was used to establish the relative level of gene transcripts based on the 2^−ΔΔCT^ method [55].

### 4.7. Hybridization of Cellular RNA with the Fluorescent Probe ROX-miR775A

The RNA interference analysis was performed using the fluorescent probe ROX-miR775A, which is a nucleotide sequence complementary to mature miR775A, with an ROX fluorophore at the 3′ end. For the analysis, 100 ng of total cellular RNA was taken. The results were assessed by electrophoresis in 1% agarose gel. SybrGreen I was used as an intercalating dye for nucleic acid electropherograms. The photoexcitation of SYBR Green I was performed by irradiation at 312 nm, and ROX emission was assessed.

The extraction of the fluorescent ROX-miR775A-mRNA complex from agarose gel was performed using the Cleanup Standard kit (Eurogen, Russia).

The sequencing of the ROX-miR775A-mRNA fluorescent complex was carried out at Evrogen CJSC.

### 4.8. Measurement of Total Enzymatic Activity

To isolate enzymes from maize leaves, the material (1 g) was homogenized in a porcelain mortar in an isolation medium at a ratio of 1:5. The isolation medium for fructose-1,6-bisphosphate aldolase and glucose-6-phosphate dehydrogenase contained 50 mM Tris-HCl, pH 7.8, 1 mM EDTA, and 3 mM MgCl_2_. All the procedures were carried out at 0–4 °C to prevent enzyme degradation. After sample preparation, the resulting mixture was centrifuged (Eppendorf Centrifuge 5805R, Eppendorf, Hamburg, Germany) for 5 min at 3000× *g* at 4 °C, and the supernatant was used for further studies.

The fructose-1,6-bisphosphate aldolase activity was measured at 240 nm by the formation of aldehyde during the reaction [56] in 50 mM Tris-HCl, pH 7.5, 1 mM EDTA, 3.5 mM hydrazine sulfate, and 1.2 mM fructose-1,6-bisphosphate. The glucose-6-phosphate dehydrogenase activity was determined at 340 nm in the medium containing 50 mM Tris-HCl, pH 7.8, 1 mM EDTA, 3 mM MgCl_2_, 0.5 mM NADP^+^, and 0.5 mM glucose-6-phosphate [57].

### 4.9. Isolation of Extracellular Plant Vesicles

The isolation of plant extracellular vesicles was carried out using the differential ultracentrifugation method. From 0.5 g of plant material, pure apoplastic fluid was collected by vacuum infiltration and centrifugation at 900× *g* for 10 min at +4 °C. The isolation of two vesicle fractions from the apoplastic fluid was performed by successive stages of low-speed centrifugation at 2000× *g* for 10 min and 10,000× *g* for 30 min at +4 °C, with subsequent high-speed ultracentrifugation at 40,000× *g* for 60 min and 100,000× *g* for 60 min at +4 °C on a Beckman LB50 centrifuge (Beckman, Brea, CA, USA) [58]. All the stages of extracellular vesicle isolation were performed at +4 °C.

The isolation of extracellular vesicles after separation into P40 and P100 fractions was assessed using the microscopy method on an Olynpus CX41 device (Olympus, Tokyo, Japan) with a magnification of 1000×. The quantitative assessment of the nucleic acid content in samples isolated from fraction P100 and their purity was performed spectrophotometrically using a NanoPhotometer C40 (Implen, Munich, Germany).

### 4.10. Inhibitory Assay

To determine the location of miRNA inside or on the surface of vesicles, the P100 fraction was treated with 10 units of proteinase K (SibEnzyme, Russia) for 15 min at 37 °C with Triton X-100. The samples were then treated with 10 units of RNase (SibEnzyme, Russia) for 15 min at 37 °C. The inhibition of enzymes after treatment was performed by heating the reaction mixture at 70 °C for 5 min [58]. Immediately after treatment with proteinase and RNase, total RNA was extracted using phenol-chloroform extraction for the further detection of specific miR775A. The quantitative assessment of the nucleic acid content in samples isolated from fraction P100 and their purity was performed spectrophotometrically using a NanoPhotometer C40 (Implen, Munich, Germany).

### 4.11. Statistical Data Processing

The experiments were conducted in three biological and four analytical replicates. The data were subjected to a two-way analysis of variance (ANOVA) using STATISTICA data analysis software version 9.0 (Statsoft Wipro, East Brunswick, NJ, USA). The results are presented as mean values and standard deviations (SD). Statistically significant differences are discussed at *p* < 0.05 [59]. The letters represent significant differences according to a one-way ANOVA analysis at *p* < 0.05 (Tukey’s multi-comparison). The electrophoregram and microscopy images represent the data from typical experiments repeated three to four times.

## 5. Conclusions

An increase in the total content of free miR775 and its participation in the suppression of the *Aldo1* gene represents an important mechanism in the regulation of glycolysis and the development of the adaptive reaction of cellular metabolism in response to hypoxia.

## Data Availability

The datasets generated for this study are available upon request from the corresponding author.

## Abbreviations

AGO1	ARGONAUTE1
EV	extracellular vesicle
miRNA	microRNA
miR775A	microRNA 775A
OPPP	oxidative pentose phosphate pathway
pri-miR	initially transcribed long primary transcripts from the precursor gene of the corresponding microRNA
pre-miR	hairpin-shaped precursor processed into microRNA
TF	transcription factor
TCA cycle	tricarboxylic acid cycle
